# Metasurfaces of capacitively loaded metallic rings for magnetic resonance imaging surface coils

**DOI:** 10.1038/s41598-023-30185-y

**Published:** 2023-02-21

**Authors:** Manuel J. Freire

**Affiliations:** Department of Electronics and Electromagnetism, Facultad de Física, University of Seville, Avenida Reina Mercedes s/n, 41012 Sevilla, Spain

**Keywords:** Electrical and electronic engineering, Applied physics

## Abstract

This work investigates the use of a metasurface made up of a two-dimensional array of capacitively loaded metallic rings to enhance the signal-to-noise ratio of magnetic resonance imaging surface coils and to tailor the magnetic near-field radio frequency pattern of the coils. It is found that the signal-to-noise ratio is increased if the coupling between the capacitively loaded metallic rings in the array is increased. The input resistance and the radiofrequency magnetic field of the metasurface loaded coil are numerically analyzed by means of an efficient algorithm termed the discrete model to determine the signal-to-noise ratio. Standing surface waves or magnetoinductive waves supported by the metasurface introduce resonances in the frequency dependence of the input resistance. The signal-to-noise ratio is found to be optimal at the frequency corresponding to a local minimum existing between these resonances.The discrete model is used in an optimization procedure to fit the structural parameters of a metasurface to enhance the signal-to-noise ratio at the frequency corresponding to this local minimum in the input resistance. It is found that the signal-to-noise ratio can be greatly improved if the mutual coupling between the capacitively loaded metallic rings of the array is made stronger by bringing them closer or by using rings of squared shape instead of circular. These conclusions derived from the numerical results provided by the discrete model are double-checked by means of numerical simulations provided by the commercial electromagnetic solver Simulia CST and by experimental results. Numerical results provided by CST are also shown to demonstrate that the surface impedance of the array of elements can be adjusted to provide a more homogeneous magnetic near-field radio frequency pattern that ultimately leads to a more uniform magnetic resonance image at a desired slice. This is achieved by preventing the reflection of propagating magnetoinductive waves at the edges of the array by matching the elements arranged at the edges of the array with capacitors of suitable value.

## Introduction

Metamaterials (MM) are artificial periodic structures made up of sub-wavelength resonant elements that have emerged in the past two decades as a new class of electromagnetic materials. MM can be engineered to achieve exotic electromagnetic properties^1^. Thus, for example, one of the most striking properties of MM is the ability of a slab with negative permittivity/permeability to behave as a lens with sub-wavelength resolution for the electric/magnetic field^1–3^. MM applications range from microwave and radiofrequency (RF) bands to optical frequencies^4^, with new functionalities in imaging technology recently explored in the optical range^5–7^. MM exhibit an inherent narrowband response due to the resonant nature of its constituent elements. This narrowband response is usually considered the main drawback of MM in different fields of application. However, this is not the case for an application with an inherent narrow band of operation such as magnetic resonance imaging (MRI), which involves the detection of RF signals ranging from tens to hundreds of MHz with bandwidths of tens of kHz^8^. Bulk MM made up of three-dimensional (3D) arrays of elements have been explored for their use in MRI in the form of RF magnetic flux guides and lenses^9–22^. Compared to bulk MM, metasurfaces (MS) made up of two-dimensional (2D) arrays are a desirable alternative, as they are thin and flexible structures that are easier to manufacture and can provide the same performance as bulk structures in certain applications^23^. In the literature, MS have been proposed for application in MRI to provide local enhancement of the signal-to-noise ratio (SNR) of MRI coils^24–28^(SNR is the main figure of merit that assesses the quality of MRI). All of these MS reported are based on arrays of conducting wires. In the present work, the application in MRI of MS made up of elements consisting of split metallic rings loaded with lumped capacitors, or capacitively loaded rings (CLR), is investigated. In previous works, the author has explored the use of bulk MM made up of 3D arrays of CLR to locally increase the SNR of surface coils^14–22^. These bulk structures were designed to behave as lenses of negative permeability with sub-wavelength resolution^14–21^ or as slabs that exhibit zero^22^ or high permeability^22^ for the RF magnetic field. In any case, these bulk structures were designed following a homogenization procedure to model a 3D array of CLR as a medium with an effective parameter (permeability)^1,15^. In the present work, a different viewpoint is required for the analysis of MS made up of 2D arrays of CLR. An MS is characterized by a surface impedance rather than an effective parameter. In addition, as will be discussed later, an MS made up of CLR supports propagating surface waves. In the present work, the ability of the MS of CLR to provide local enhancement of the SNR of surface coils is investigated, and a key point of this investigation is the role that the excitation of standing surface waves plays in the frequency dependence of the SNR. Moreover, the possibility of tailoring the near-field of an MS-loaded coil by modulating the impedance of the CLR is also investigated, as well as the role played once again by the excitation of surface waves. In particular, it will be shown that, by adding a suitable terminal impedance in all the CLR arranged along the edges of the array, the reflection of surface waves at the edges of the array is prevented and this leads to a more homogeneous field pattern at certain distance. This ultimately allows one to obtain a more uniform MRI image in a desired slice, which is of great interest in MRI.

**Figure 1:**
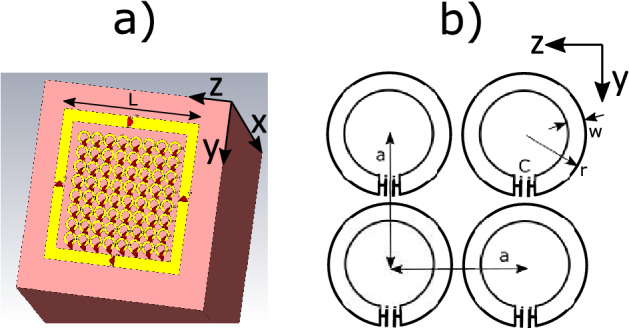
(**a**) Picture of the configuration to be analyzed in this work: a squared surface of length *L* loaded with a 2D CLR array and a cubic conducting sample resembling human tissue. (**b**) Schematic of the array of CLRs with periodicity *a*, external radius of the ring *r*, strip width *w* and capacitance of the lumped capacitor *C*.

Figure 1.a shows a picture of the configuration used in this work to carry out the investigation mentioned above: a squared surface coil loaded with a 2D array of CLR and a conducting sample that resembles human tissue. The picture corresponds to an implementation in the commercial electromagnetic solver Simulia CST, which is used in this work, as described later. The square coil is split and capacitors are inserted into the gaps to homogenize the current, as usual in the designs of MRI coils^29^. Figure 1.b shows a schematic of the CLR array together with geometric parameters.

In general, an MRI coil is electrically modeled as a circuit consisting of a series inductance with resistance. This resistance is the input resistance, $R_\text {in}$, introduced into the coil by the ohmic losses of the sample or tissue^8^. In general, the SNR provided by the coil is proportional to the ratio between the sensitivity, or the RF magnetic field per unit current produced by the coil$, B_1$, as specifically noted in MRI, and the square root of $R_\text {in}$^8^:

1 $$\begin{aligned} \text {SNR}\propto \frac{B_1}{\sqrt{R_\text {in}}} \end{aligned}$$

This magnetic field produced by a single coil is linearly polarized. To obtain circular polarization, two coils are required, arranged with their axes perpendicular to each other, and fed in phase quadrature^8^. For the configuration shown in Fig. 1.a, $B_1$ corresponds to the total field produced by the coil and the CLR array. The CLR array can enhance the field of the coil but also introduces additional losses in the coil. Thus, $R_\text {in}$ for the configuration of Fig. 1.a corresponds to the losses introduced in the coil by both the sample and the CLR array. The contribution to $R_\text {in}$ of ohmic losses due to coil metallization can be neglected in MRI systems for operation frequencies corresponding to high field values (1 T to 7 T) and ultra-high ($\ge 7$ T)^8^. In the present work, the study is carried out for a configuration operating at the typical frequency of a 1.5 T MRI system, which is 63.6 MHz, and the ohmic losses associated with the copper coil can be neglected. The SNR provided by the configuration coil in Fig. 1.a can then be enhanced by the CLR array if the total field is maximized and, at the same time, the additional input resistance introduced by the CLR array is minimized. In the present work, the structural parameters of the CLR array are optimized to achieve both goals.

The use of small parasitic loops magnetically coupled to a receiving surface coil to locally improve coil sensitivity has been explored since the early days of MRI^30^. More recently, the use of arrays of passive loops magnetically coupled to a volume coil has also been explored in high-field systems to locally enhance the transmit field of a birdcage coil and thus to homogenize the total transmit field^31^. In any case, since parasitic or passive loops are magnetically coupled with the active coil (receiver or transmitter coil) through an axial configuration, the mutual inductance between them is positive. Thus, the currents induced in the passive loops by Faraday’s law are in phase with the current in the active coil if the reactance of the passive loops is negative or capacitive. This happens at frequencies below the frequency of resonance of the loops. Therefore, below the frequency of resonance, the fields of the passive loops and the coil are added, and the sensitivity is enhanced. In the present work, the CLR in the configuration under study are also designed to work below their resonance frequency to behave capacitively. The CLRs are also magnetically coupled between them, and therefore the array supports the waves due to voltages induced by time-varying magnetic fields or magnetoinductive (MI) waves^32,33^. Since the CLR of the array in Fig. 1 are arranged in a coplanar configuration, the supported MI waves are backward^32,33^. For the study shown in this work, it is worth noting that, due to the finite size of the array, standing MI waves can be excited with wavelengths that are proportional to the size of the array.

In the present work, the study of the configuration shown in Fig. 1.a is carried out using a dedicated algorithm developed for the analysis of coils in the presence of CLR arrays and a half-space of conductance similar to that of human tissue^19^. This algorithm was developed for the numerical analysis of MM, taking into account the discrete and finite nature of the structure. From now on, this algorithm will be termed a discrete model in the text. With this discrete model, the value of $R_\text {in}$ and the spatial dependence of $B_1$ along the coil axis are calculated for the configuration shown in Fig. 1.a with different structural parameters. Since the SNR is proportional to the ratio $B_1/\sqrt{R_\text {in}}$, as stated in expression (1), and it is of interest for the analysis to compare the SNR obtained for different structures, for the present analysis, the SNR will be considered not proportional but equal to this ratio. Calculating $B_1$ and $\sqrt{R_\text {in}}$ with the discrete model is very quick compared to the use of commercial electromagnetic solvers. This allows us to study the dependence of the SNR along the coil axis with the structural parameters of the CLR array in an efficient optimization procedure. In the discrete model, a array equation for unknown currents in the CLR and coil is solved^19^. The diagonal elements of the impedance array correspond to the self-impedances of the CLR and the off-diagonal elements correspond to the mutual inductances between the CLR. The study carried out with the analysis of different structures with the discrete model shows that the value of $R_\text {in}$ introduced by the CLR array exhibits resonances at frequencies corresponding to the excitation of standing MI waves. Furthermore, the optimal SNR is obtained at the frequency that corresponds to a local minimum of $R_\text {in}$ between these resonances. A significant outcome of the study is the fact that as the mutual coupling between the CLR increases, the value of this local minimum of $R_\text {in}$ is reduced, and consequently the SNR increases. In practice, this is achieved by reducing the periodicity of the array. Thus, one of the main conclusions of the present work is the finding that the SNR of a surface MRI coil can be enhanced by means of MS of strongly coupled elements rather than weakly. Once an optimal structure is determined from the point of view of the SNR along the coil of the axis with the discrete model, the 3D magnetic field pattern is obtained by using the commercial electromagnetic solver Simulia CST and compared with the field produced by the coil in the absence of the array for comparison purposes. A square coil and a CLR array are manufactured with the optimal structural parameters and experimental results are obtained to validate the comparison mentioned above. These experimental results are obtained by measuring with a vector network analyzer (VNA) the transmission coefficient $S_{21}$ between the squared coil and a small probe immersed inside a saline solution phantom, both in the presence and absence of the intermediate CLR array placed between the coil and the phantom. The coil is matched to 50 $\Omega$ in each case (see the Appendix for a demonstration of the relation between the transmission coefficient $S_{21}$ for a matched coil and the SNR). As has been pointed out, the main conclusion of the study is that increasing the mutual coupling between CLR results in an increase of the SNR and that this can be achieved in practice by bringing the CLR closer, that is, by reducing the periodicity. Moreover, it is also shown that once the periodicity cannot be more reduced for a given size of the CLR, a stronger coupling can still be obtained and, therefore, a higher increase in the SNR can be achieved by using squared rings instead of circular rings. Finally, numerical results provided by Simulia CST are also shown to demonstrate that the RF magnetic field pattern produced by a CLR array can be tailored, for example, to produce a homogeneous field pattern at a plane at a desired distance and, therefore, to obtain a homogeneous MR image at a desired slice. This is achieved by considering a remedy used to avoid reflections of MI waves in a 1D array of resonators of finite length and that consists of adding a terminal impedance at the ends of the 1D array^32,33^. This remedy is extended to the present case of a 2D CLR array. In the present work, the reflections at the edges of the 2D CLR array of the MI waves propagating in the structure are suppressed by matching the CLR arranged along the edges of the array with a suitable capacitance value.

The paper is structured as follows. In Sect. “MI waves and the input impedance of a coil loaded with a CLR array”, it is analyzed the role that MI waves excited by a coil in finite-size CLR arrays play in the frequency dependence of $R_\text {in}$. The standing MI waves supported by the array are shown to lead to resonances in $R_\text {in}$, with a local minimum for $R_\text {in}$ between these resonances. In Sect. “MI waves and the SNR of a coil loaded with a CLR array”, the dependence of the SNR value with the frequency dependence of $R_\text {in}$ is analyzed. In Sect. “A stronger CLR coupling reduces $R_\text {in}$ and enhances SNR”, a result of very practical interest is demonstrated, which is the fact that the SNR can be enhanced by increasing the mutual coupling between CLR in the array, and that this can be achieved by bringing the CLR closer to each other or by using squared elements rather than circular. In Sect. “Tailoring the near field”, an example of a CLR array design is shown which provides a more homogeneous field pattern. This is achieved by suppressing the reflection of MI waves at the edges of the array. Conclusions are drawn in Sect. “Conclusion”. In Appendix “Linear relation between SNR and S$_{21}$”, the relation between SNR as defined in expression (1) and the transmission coefficient or scattering parameter $S_{21}$ measured with a VNA is demonstrated.

## MI waves and the input impedance of a coil loaded with a CLR array

**Figure 2:**
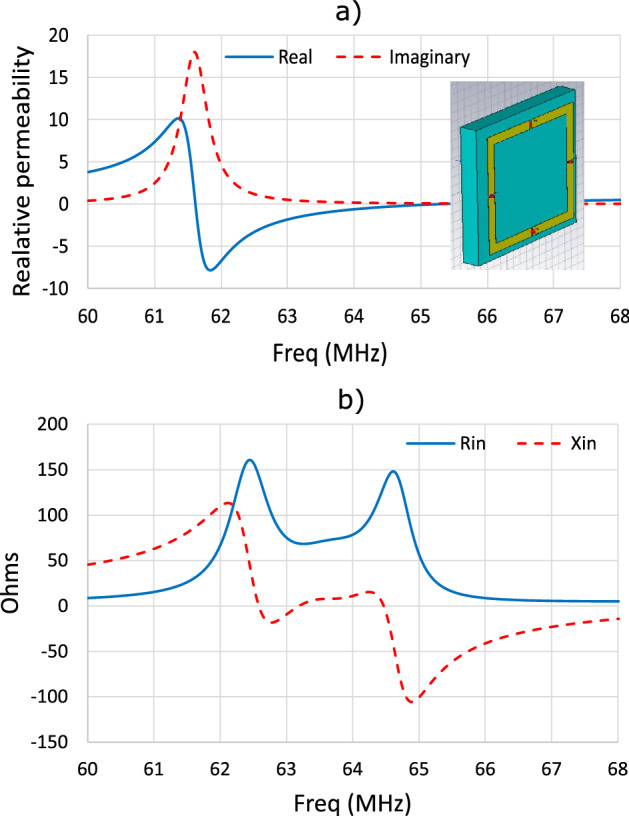
(**a**) Frequency dependence of the real and imaginary parts of the complex permeability $\mu$ of a homogeneous MM slab with the dispersive parameters of a MM lens previously analyzed by the authors^14–16^. The dispersion parameters of $\mu$ are chosen to obtain $\mu = -1 -j 0.2$ at 63.6 MHz. Inset: picture of a 12-cm squared coil loaded with the MM slab of dimensions $15 \times 15 \times 1.8$ cm^3^. (**b**) Frequency dependence of the input resistance $R_\text {in}$ and the input reactance $X_\text {in}$ of the coil in the presence of the MM slab obtained with Simulia CST.

For the analysis, it is helpful to previously discuss the frequency dependence of the input impedance of a coil loaded with a homogeneous slab of MM with negative permeability ($\mu <0$), and then establish an analogy with the frequency dependence of the input impedance of a similar coil loaded with a CLR array. Figure 2.a shows the frequency dependence of the real and imaginary parts of $\mu$ for a homogeneous slab of MM implemented in Simulia CST for its simulation. The figure shows in the inset the screenshot of the simulation structure implemented that corresponds to a slab $15 \times 15 \times 1.8$ cm^3^ and a squared coil of 12 cm in length and 1 cm in width of the strip. In the simulation, three 154 pF capacitors are evenly distributed in three gaps along the strip to homogenize the current in the coil, and a port is placed in the fourth gap. The dispersion parameters of $\mu$ are chosen to obtain $\mu = -1 -j 0.2$ at 63.6 MHz, that is, the operating frequency of a 1.5 T MRI system. This complex value of $\mu$ corresponds to that of a MM lens previously analyzed by the author^14–16^. Figure 2.b shows the results provided by Simulia CST for the frequency dependence of the input resistance $R_\text {in}$ and the input reactance $X_\text {in}$ introduced by the MM slab in the coil. $R_\text {in}$ shows two maximums at frequencies that correspond to the excitation of magnetostatic waves on the slab. Between these two maximums, at the frequency that corresponds to the real part of $\mu$ equal to $-1$ (63.6 MHz), $R_\text {in}$ shows a minimum and $X_\text {in}$ cancels.

**Figure 3:**
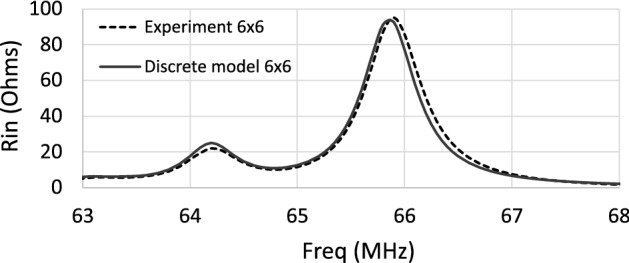
$R_\text {in}$ measured and calculated for a 12 cm long square coil loaded with an array of $6 \times 6$ CLR. Structural parameters in the experiment: *r* = 6 mm, *w* = 2 mm, *a* = 15 mm, *C* = 470 pF $\pm 1 \%$. Structural parameter in the discrete model: *r* = 6 mm, *w* = 2 mm, *a* = 15.6 mm, *C* = 473 pF. The array is placed 6 mm from the coil.

In view of the above, it is now interesting to discuss the frequency dependence of the input impedance for a similar coil loaded with a CLR array. For simplicity, the discussion will focus on the analysis of only the values of $R_\text {in}$. To do this, a numerical analysis is performed that makes use of the discrete model mentioned in the Introduction Section^19^. For the sake of simplicity, follow-up analysis in this section will be carried out in the absence of the conducting sample. The discrete model has already been validated previously for the calculation of $R_\text {in}$^19^. However, at this point, a comparison is made between the calculation with the discrete model and the measurement of $R_\text {in}$ for a particular structure, to consider this comparison as a reference to the accuracy of the present study. Thus, Fig. 3 shows the value of $R_\text {in}$ measured for a 12 cm long squared coil, in the absence of a conducting sample or phantom, loaded with an array of $6 \times 6$ circular CLR fabricated with the following structural parameters: *r*= 6 mm, *w* = 2 mm, *a* = 15 mm and capacitors by Passive Plus with $C=$ 470 pF and tolerance $\pm 1 \%$. The distance between the coil and the array is 6 mm. Figure 3 also shows the numerical results provided by the discrete model for $R_\text {in}$, which are totally in agreement with the experimental results. The numerical results shown in Fig. 3 are obtained after fitting the following parameters in the discrete model to these values: $a=15.6$ mm, $C=473$ pF.

**Figure 4:**
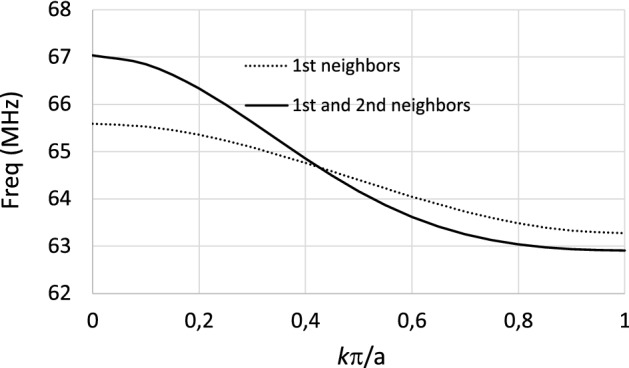
Dispersion relation calculated for the array analyzed in Fig. 3 with $r=6$ mm, $w=2$ mm, $a=15$ mm and $C=470$ pF, in the approximation of the interactions of the first neighbors and in the approximation of the interactions of the first and second neighbors.

While the peaks shown in Fig. 2 in the frequency dependence of $R_\text {in}$ for the homogeneous MM slab correspond to the excitation of magnetostatic waves, the peaks shown in Fig. 3 for the CLR array correspond to the excitation of standing MI waves whose wavelength fits the finite size of the array. Once the precision of the discrete model has been established by the agreement shown in Fig. 3, the discrete model is used to study the frequency dependence of the position of the peaks or resonances in $R_\text {in}$ with the size of the array, that is, with the number of elements along the length of the array. Figure 4 shows the dispersion relation calculated^32^ for the array analyzed in Fig. 3 for $a=15$ mm and $C=470$ pF, in the approximation of the interactions of the first neighbors and in the approximation of the interactions of the first and second neighbors.

**Figure 5:**
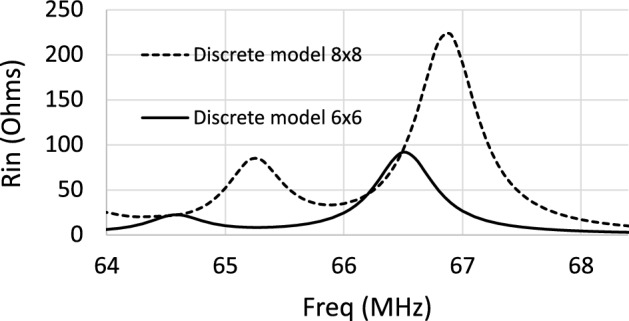
Calculations provided by the discrete model for the values of $R_\text {in}$ in a 12 cm square coil loaded with CLR arrays with $r=6$ mm, $w=2$ mm, $a=15$ mm, $C=470$ pF and different number of elements: $6 \times 6$ and $8 \times 8$ unit cells. Each array is placed at 6 mm from the coil.

As mentioned above, the peaks or resonances in $R_\text {in}$ for a CLR array correspond to the excitation of standing MI waves associated with different wavelengths in the dispersion relation of MI waves. Note that, since the RF magnetic field pattern produced by the coil that excites the array is maximum at the center of the array, the standing MI waves that can be excited by the coil and that lead to the peaks in $R_\text {in}$ must correspond to standing MI waves with an odd number of half-wavelengths throughout the length of the array. From the dispersion relation shown in Fig. 4, in the approximation of the interactions between the first and second neighbors, Table 1 shows the values of the frequencies in MHz that correspond to the normalized wavenumber $k \pi /a$ such that a half-wavelength or three half-wavelengths fits the length of an array of $6 \times 6$ unit cells or $8 \times 8$ unit cells. Figure 5 shows the calculation provided by the discrete model for the frequency dependence of $R_\text {in}$ in the 12 cm long square coil loaded with an array of $6\times 6$ unit cells or $8 \times 8$ unit cells with the same structural parameters as in the dispersion relation shown in Fig. 4. Thus, Table 1 also shows the frequency values in MHz for the position of the peaks or resonances in $R_\text {in}$ in the curves from Fig. 5. The agreement shown in Table 1 between the frequency values for the peaks or resonances provided by the discrete model in the calculation of $R_\text {in}$ and the frequency values provided by the dispersion relation confirms that the resonances in $R_\text {in}$ correspond to the excitation of standing MI waves such that half-wavelength or three half-wavelengths of the standing wave fit the length of an array of $6 \times 6$ unit cells or $8 \times 8$ unit cells. Note that this agreement requires one to consider both first- and second-neighbor interactions.

**Table 1:** Frequency values in MHz for the corresponding wavenumbers in the dispersion relation of Fig. 4 in the approximation of both the first and second neighbors such that a half-wavelength or three half-wavelengths fits the length of an array of $6 \times 6$ unit cells or $8 \times 8$ unit cells, and frequency values in MHz for the peaks or resonances in $R_\text {in}$ of Fig. 5.

$6 \times 6$ units cells	Dispersion relation	Resonance in R$_\text {in}$
$\lambda /2=6a \Longrightarrow ka/\pi =1/6=0.166$	66.6 MHz	66.5 MHz
$3\lambda /2=6a \Longrightarrow ka/\pi =3/6=0.500$	64.6 MHz	64.6 MHz
$8 \times 8$ units cells	Dispersion relation	Resonance in R$_\text {in}$
$\lambda /2=8a \Longrightarrow ka/\pi =1/8=0.125$	66.8 MHz	66.9 MHz
$3\lambda /2=8a \Longrightarrow ka/\pi =3/8=0.375$	65.3 MHz	65.3 MHz

Furthermore, it is worth noting also that Fig. 5 shows that as the size of the array increases from $6\times 6$ to $8 \times 8$ unit cells, the peaks move to higher frequencies. This is due to the backward characteristic of the dispersion relation, which is clearly shown in Fig. 4. As the size of the array increases from $6\times 6$ to $8 \times 8$, the standing MI waves that can resonate within the size of the array have larger wavelengths, so smaller wavenumbers, and this corresponds to higher frequencies in the backward dispersion relation.

## MI waves and the SNR of a coil loaded with a CLR array

**Figure 6:**
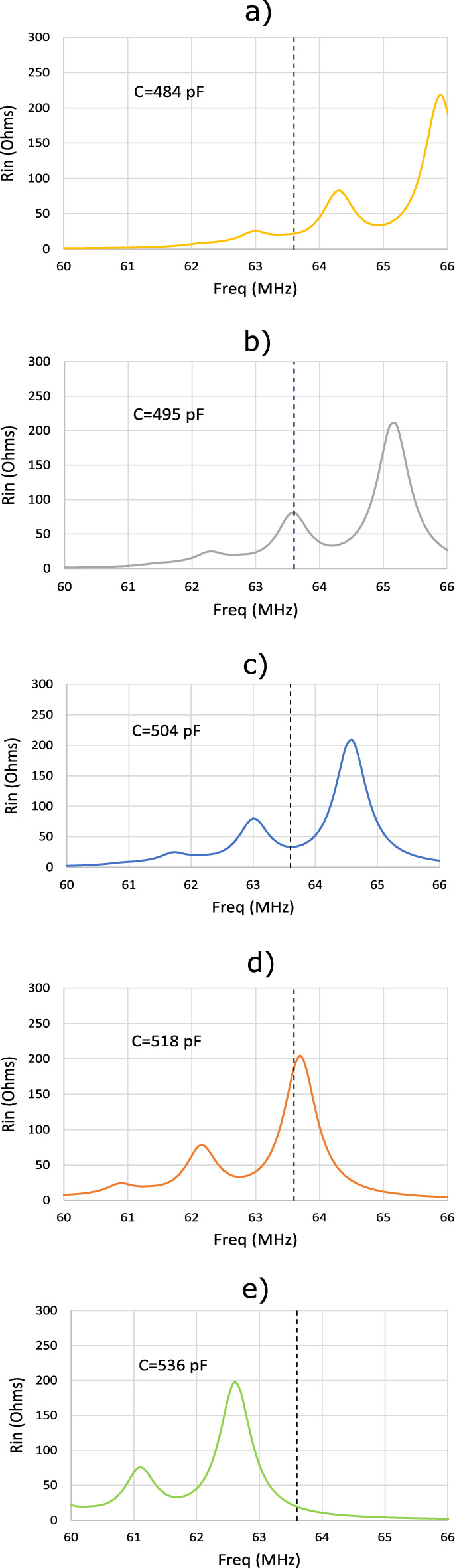
Calculations provided by the discrete model for the values of $R_\text {in}$ of a 12-cm square coil in length loaded with $8 \times 8$ CLR arrays with $r=6$ mm, $w=2$ mm, $a=15$ mm and different values of *C*: (**a**) $C=484$ pF, (**b**) $C=495$ pF, (**c**) $C=504$ pF, (**d**) $C=518$ pF, (**e**) $C=536$ pF. The dashed vertical lines indicate the frequency of 63.6 MHz that corresponds to the operating frequency of a 1.5T MRI system. Each array is placed 6 mm from the coil.

**Figure 7:**
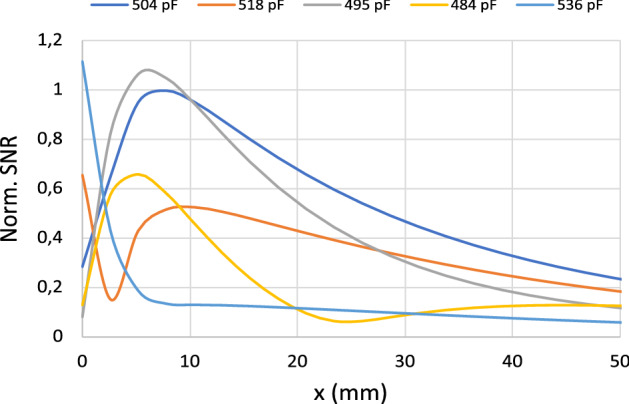
Calculations provided by the discrete model for the SNR along the axis of a 12 cm long square coil loaded with arrays of $8 \times 8$ CLR with $a=15$ mm, $r=6$ mm, $w=2$ mm and different values of the *C* that correspond to the different values indicated in Fig. 6: 484 pF, 495 pF, 504 pF, 518 pF, 536 pF. All curves are calculated for the same frequency of 63.6 MHz that corresponds to the operating frequency of a 1.5 T MRI system. Each array is placed at 6 mm from the coil and the array is 6 mm far from a conducting half-space with $\sigma =0.7$ S/m and $\varepsilon =70$.

Once it has been demonstrated that the peaks in $R_\text {in}$ already correspond to standing MI waves with half-wavelength or three half-wavelengths resonating throughout the array, it is next checked that the SNR provided by the coil in the presence of the array is optimal at the frequency corresponding to the local minimum of $R_\text {in}$ between the peaks. To carry out this study, the discrete model is used to calculate from the expression (1) the SNR along the axis of the 12 cm long square coil loaded with an array of $8 \times 8$ elements similar to that previously analyzed in Fig. 5, and in the presence of a half-space with conductivity $\sigma =0.7$ S/m and dielectric constant $\varepsilon =70$. For comparison purposes, this calculation is performed for values of $R_\text {in}$ that correspond to the peaks and to the local minimum between the peaks, and, for practical interest, by fixing the calculation frequency so that the value of interest of $R_\text {in}$ is always fixed for each case at 63.6 MHz, which corresponds to the working frequency of a 1.5T MRI. This is achieved by tuning the value of *C* for each calculation. Figure 6 shows the calculated frequency response of $R_\text {in}$ for several values of *C*. A vertical dashed line highlights for each case the frequency of 63.6 MHz, and points out the position of a peak in $R_\text {in}$ for $C=495$ and $C=518$ pF, and the minimum between peaks for $C=504$ pF. For comparison purposes, two additional values of $C=484$ pF and $C=536$ pF are also chosen to perform SNR calculations beyond the peaks. Figure 7 shows the results of the SNR calculation provided by the discrete model along the coil axis at a frequency of 63.6 MHz for the different capacitance values of Fig. 6. It must be noted that in Fig. 7, the curve for $C=504$ pF is above all for almost the whole distance range along the coil axis, indicating that the SNR is optimal for this case, which corresponds to the situation depicted in Fig. 6.c, that is, with the coil operating at 63.6 MHz at a minimum of $R_\text {in}$ between the peaks.

## A stronger CLR coupling reduces $R_\text {in}$ and enhances SNR

In previous sections, it has been demonstrated that a CLR array introduces resonances in the input resistance, $R_\text {in}$, of a coil, and that the SNR provided by the coil in the presence of the array is optimal at the frequency corresponding to the minimum value that $R_\text {in}$ takes between these resonances. Once this has been established, an optimization procedure is followed based on the structural parameters of the array to reduce the magnitude of the minimum value of $R_\text {in}$ and therefore increase the SNR. The discrete model is used to calculate the frequency dependence of $R_\text {in}$ for a 12 cm square coil loaded with an array, starting from the structure of $8 \times 8$ elements analyzed in Figs. 6 and 7, and tuning the values of *a* and *C* to fix the minimum value of $R_\text {in}$ at a frequency of 63.6 MHz, and at the same time to reduce the magnitude of this minimum.

**Figure 8:**
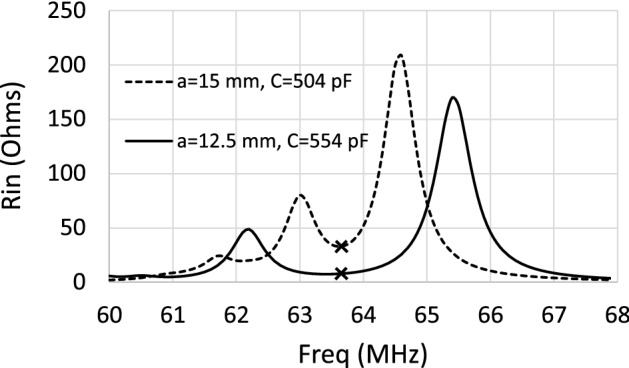
Calculations provided by the discrete model for the value of $R_\text {in}$ for a 12 cm square coil in length loaded with two different arrays of $8 \times 8$ CLR with the same size ($r=$6 mm, $w=2$ mm) but different values of *a* and *C*: $a=15$ mm, $C=504$ pF (solid line); $a=12.5$ mm, $C=554$ pF (dashed line). The crosses in the curves point to the frequency of 63.6 MHz. Each array is placed 6 mm from the coil.

**Figure 9:**
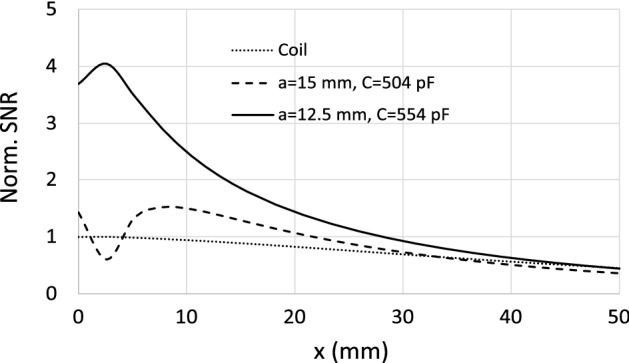
Calculations provided by the discrete model at 63.6 MHz for the SNR along the axis of a 12 cm long squared coil (dotted line) loaded with each of the $8 \times 8$ CLR arrays analyzed in Fig. 8: $a=15$ mm and $C=504$ pF (dashed line); $a=12.5$ mm and $C=554$ pF (solid line). In both cases $r=6$ mm and $w=2$ mm. Each array is placed at 6 mm from the coil and the array is at 6 mm from a conducting half-space with $\sigma =0.7$ S/m and $\varepsilon =70$. The results are normalized to the value obtained at $x=0$ in the absence of arrays (dotted line).

Figure 8 shows a comparison between the frequency dependence of $R_\text {in}$ for the starting structure with $a=15$ mm and $C=504$ pF and the final structure obtained after the optimization procedure with $a=12.5$ mm and $C=554$ pF. It must be noted that, although this optimized structure has a reduced periodicity (from 15 mm to 12.5 mm), the CLR are of the same size, and despite this reduction of the periodicity, the CLR in the optimized array are not in touch. The curves in Fig. 8 show that the minimum value of $R_\text {in}$ at the frequency of 63.6 MHz between the peaks (marked with $\times$ in the curves of the figure) is considerably reduced for the optimized structure. In particular, the value of $R_\text {in}$ is reduced from 34 $\Omega$ to 8 $\Omega$. As it was explained in the Introduction Section, the enhancement of the SNR comes from the reduction in the value of $R_\text {in}$ and from the addition of the fields produced by the coil and the CLR. This last condition is fulfilled if the CLR operate at a frequency below the frequency of resonance, as was also pointed out. In the array analyzed above with $a=12.5$ mm, for the nearest neighbors in the same row, the discrete model provides a value for the mutual inductance of $M_\text {r}=-0.48$ nH and for the nearest neighbors on the same diagonal, a value of $M_\text {d}=-0.13$ nH. These values are negative since they correspond to the mutual inductances of CLR arranged in coplanar configuration. The self-inductance of an isolated CLR is 13.45 nH, calculated from the values of the external radius and the width of the strip using a known model^34^. Thus, mutual inductance with all nearest neighbors reduces the self-inductance to a value of 11.01 nH, and with $C=554$ pF this provides a resonance frequency of 64.4 MHz. Therefore, the CLR actually behave capacitively since the operation frequency of 63.6 MHz is below the resonance frequency of 64.4 MHz. Taking this into account, and the significant reduction in $R_\text {in}$ from 34 $\Omega$ to 8 $\Omega$ shown by the results in Fig. 8, the reduction of the periodicity from $a=15$ mm to $a=12.5$ mm should lead to a clear enhancement of the SNR as established by expression (1). To check this, Fig. 9 shows the calculation carried out with the discrete model for the SNR along the coil axis at 63.6 MHz for each of the two arrays analyzed in Fig. 8, in the presence of a conducting half-space with $\sigma =0.7$ S/m and $\varepsilon =70$. The results shown in Fig. 9 demonstrate that the axial SNR is significantly enhanced for the CLR array with reduced periodicity $a=12.5$ mm.

**Figure 10:**
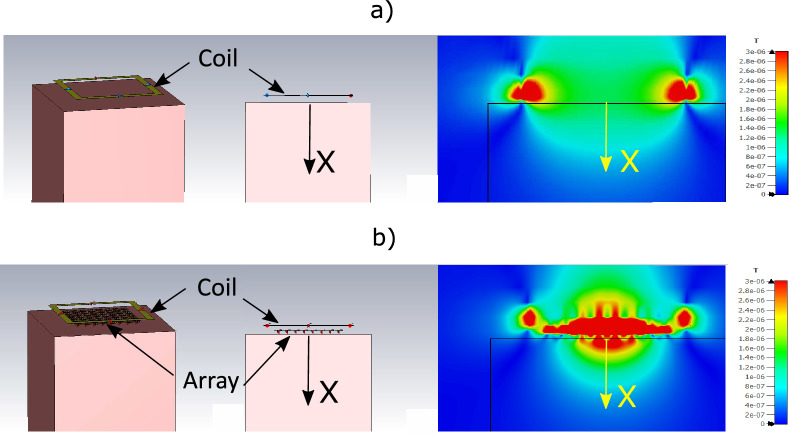
Screenshots of a perspective view and a front view of the configurations under analysis together with the RMS B$^+_1$ maps provided by Simulia CST for (**a**) a squared coil of 12 cm in length placed at 6 mm from a cubic sample resembling human tissue with $\sigma =0.7$ S/m and $\varepsilon =70$ and (**b**) the same coil loaded with an array of $8 \times 8$ CLR with parameters $r=$6 mm, $w=2$ mm, $a=12.5$ mm, $C=525$ pF, the array being placed placed at 6 mm from the coil and the cubic sample at 6 mm from the array. The coil is matched to 50 Ohms in each case trough the corresponding circuit matching network.

Because reducing the periodicity while maintaining the size of the CLRs must lead to an increase in the mutual inductance between neighboring CLRs, it can be concluded that strengthening the coupling between elements in the array leads to an enhancement of the SNR. This can be considered one of the main conclusions of the present work. As a double check, the results shown in Fig. 9 have been verified using the numerical results obtained with Simulia CST. As mentioned, a commercial electromagnetic solver such as Simulia CST is very time consuming compared to the discrete model, if we are interested in computing only $R_\text {in}$ and the SNR along the coil axis as part of an optimization procedure. However, once an optimal structure has been found with the discrete model, it is of interest to use software such as Simulia CST to obtain the 3D field pattern of the RF magnetic field produced by the optimal configuration. In MRI, the average effective RF magnetic field generated by a coil is noted as $B_1^+$, and corresponds to the contribution of the longitudinal (on the axis of the coil) and transverse components of the RF field produced by the coil that are perpendicular to the static magnetic field, $B_0$, of the MRI system^8^. In MRI, $B_0$ is oriented along the z axis in a Cartesian coordinate system, and the longitudinal and transverse components of the RF field coil that are perpendicular to $B_0$ are noted as $B_{1x}$ and $B_{1y}$, respectively (the sketch depicted in Fig. 1 is in accordance with this). $B_1^+$ is a circularly-polarized field that rotates in the same direction as nuclear precession and is expressed as $B_1^+=B_{1x}+jB_{1y}$. Thus, Fig. 10 shows screenshots of the configurations under analysis implemented in CST together with the maps obtained in the simulations for the root-mean-square (RMS) value of $B_1^+$. For comparison purposes, these configurations correspond to the coil of 12 cm in length in the absence (Fig. 10a) and in the presence (Fig. 10b) of the optimized array of $8 \times 8$ elements with $a=12.5$ mm. In both cases there is a cubic conducting phantom that resembles human tissue with conductivity $\sigma =0.7$ S/m and a dielectric constant $\varepsilon =70$. The separation distance between the coil, the array and the phantom is 6 mm. Also, in both cases the coil is matched to 50 $\Omega$ through the corresponding circuit matching network. To conform to reality, the metallic pads of the real capacitors have been implemented in the CST model. This leads to an increase in the inductance of the total metallic path of the CLR. For this reason, to maintain the results at the same frequency of 63.6 MHz, the value of $C=554$ pF used with the discrete model must be reduced to $C=525$ pF in the CST simulation. The results show that the field is highly enhanced by the presence of the array with $a=12.5$ mm up to penetration depths of 3 cm inside the conducting sample.

**Figure 11:**
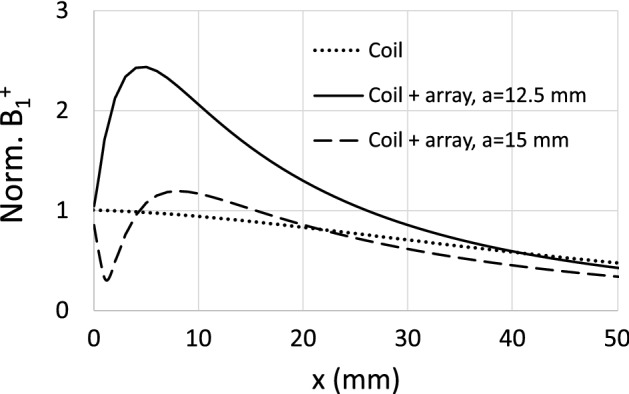
RMS B$^+_1$ profiles provided by Simulia CST for a squared coil of 12 cm in length (dotted line) placed at 6 mm from a cubic sample resembling human tissue with $\sigma =0.7$ S/m and $\varepsilon =70$ and the same coil loaded with two different arrays of $8 \times 8$ CLRs with $a=15$ mm and $C=497$ pF (dashed line); $a=12.5$ mm and $C=525$ pF (solid line). In both cases $r=$6 mm and $w=2$ mm. Each array is placed 6 mm from the coil and the cubic sample 6 mm from the array. The coil is matched to 50 Ohms in each case through the corresponding circuit matching network. The results are normalized to the value at the origin for the curve corresponding to the coil without any array.

**Figure 12:**
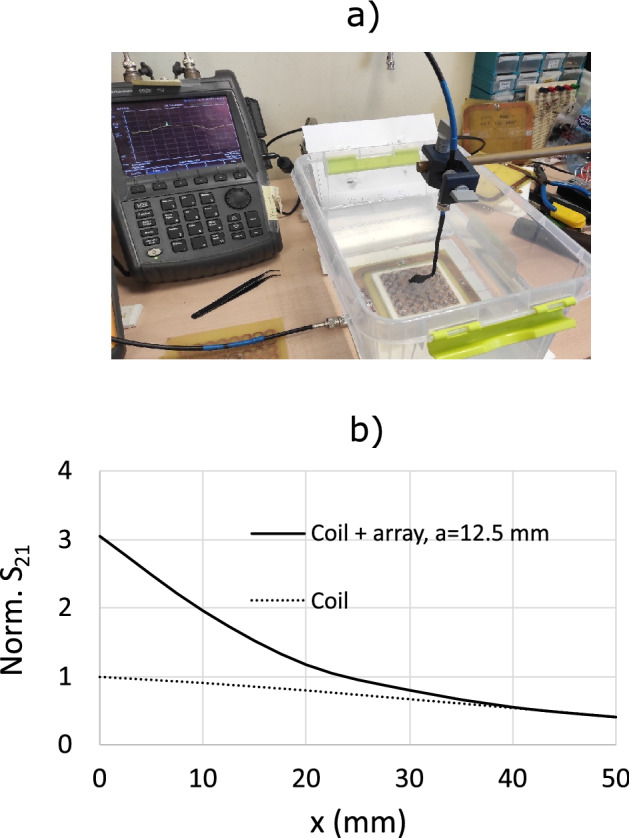
Transmission coefficient $S_{21}$ measured with a VNA between a small loop probe and a fabricated squared coil 12 cm in lenght matched to 50 $\Omega$. Measurements are carried out along the coil axis by immersing the probe inside a saline solution tank (with $\sigma =0.7$ S/m and $\varepsilon =70$, that is, the same parameters as in Fig. 10), both in the presence and in the absence of a fabricated array of $8 \times 8$ CLR with the following parameters: $r=$6 mm, $w=2$ mm, $a=12.5$ mm, $C=560$ pF. Dotted line: coil in the absence of the array. Solid line: coil in the presence of the array. The results are normalized to the measurement in the absence of the array. Comparison of $S_{21}$ in both cases is equivalent to a comparison of SNR, as demonstrated in the Appendix. The results are normalized to the value at the origin of the curve corresponding to the coil without array.

For ease of comparison, Fig. 11 shows the profiles of $B_1^+$ along the coil axis for the maps shown in Fig. 10, which correspond to an array with $a=12.5$ mm, and also for the values obtained for the array with $a=15$ mm. The curves are normalized to the value obtained at $x=0$ mm in the absence of the arrays. The profiles in Fig. 11 clearly show that there is a gain in the field provided by the array with $a=12.5$ mm compared to the field provided by the coil itself without the array and compared with the field provided by the array with $a=15$ mm. It should be noted that this gain is similar to the gain in SNR shown by the results provided by the discrete model in Fig. 9. The agreement in this gain starts from an axial distance of a few millimeters. For lower distances, the differences between the results in Figs. 11 and 9 can be attributed to the artifact produced by the local field of the individual CLR, which are modeled as a pair of wire loops in the discrete model^19^ and are more realistically modeled in the implementation in Simulia CST. For the sake of completeness, an experiment is also carried out to verify the conclusion derived from the results provided by both the discrete model and the solver Simulia CST. The experiment consists of measuring with a VNA the transmission coefficient or scattering parameter $S_{21}$ between a 12 cm long squared coil, matched to 50 $\Omega$, and a small loop probe. Measurements are carried out in the presence and absence of a fabricated $8 \times 8$ CLR array with $a=12.5$ mm. Figure 12.a shows a photograph of the experimental setup. To carry out the measurements, the probe is immersed inside a saline solution tank with the same electrical parameters as the conducting half-space in the calculations carried out with the discrete model and the conducting block in the simulations with Simulia CST. The array was fabricated by inserting capacitors by Passive Plus with a nominal value of 560 pF, which is the commercial value that is closer to the value used in the CST simulations.

**Figure 13:**
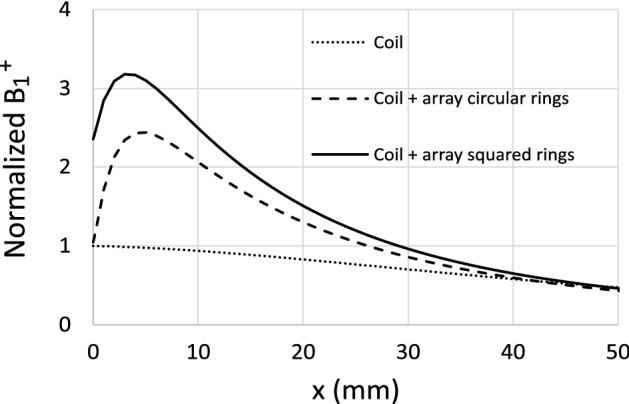
Dotted line: numerical results calculated with Simulia CST for the $B_1^+$ field of a 12 cm in length squared coil along the coil axis in the presence of a cubic sample resembling human tissue with parameters $\sigma =0.7$ S/m and $\varepsilon =70$. The distance between the coil and the sample is 6 mm. Dashed line: An array of $8 \times 8$ circular CLR with $r=$6 mm, $w=2$ mm, $a=12.5$ m, $C=525$ pF is interposed between the coil and the sample, the distance between the coil and the array is 6 mm. Solid line: an array with the same geometric parameters as in the previous case but made of squared CLR. In all cases, the coil is matched to 50 $\Omega$ through the suitable circuit matching network, and the results are obtained at a frequency of 63.6 MHz. The results are normalized to the value at the origin ($x=0$) of the curve corresponding to the coil without any array (dotted line).

Figure 12b shows the results for the measurement of the $S_{21}$ along the coil axis, both in the presence and in the absence of the CLR array. The curves are normalized to the value measured at $x=0$ mm in the absence of the array. These normalized results for $S_{21}$ can be compared with normalized results for the SNR as defined by expression (1) since both magnitudes are proportional under certain conditions, as demonstrated in the Appendix. Therefore, the experimental results in Fig. 12b can be compared with the numerical results shown in Figs. 9 and 11. Actually, the comparison between these results shows an agreement from a distance of a few mm, where the artifact produced by the individual CLR is no longer significant. In summary, in the present section, it has been demonstrated by means of calculations provided by the discrete model, simulations provided by Simulia CST, and experimental results, that the reduction of periodicity *a* in the CLR array provides an enhancement of the SNR due to the strengthening of the mutual coupling between CLR. This suggests the possibility of enhancing the SNR even more by other strategies that act on the geometry of the array. For example, a stronger coupling between CLR can be achieved by using square CLR instead of circular ones while keeping the size of the CLR and the periodicity. Thus, Fig. 13 shows the numerical results provided by Simulia CST for the field B$^+_1$ along the coil axis for the array of $8 \times 8$ circular CLR with $a=12.5$ mm, previously analyzed, and compares with the results obtained for a similar array of squared CLR. The comparison shows an increase in the field for the latter case.

## Tailoring the near field

**Figure 14:**
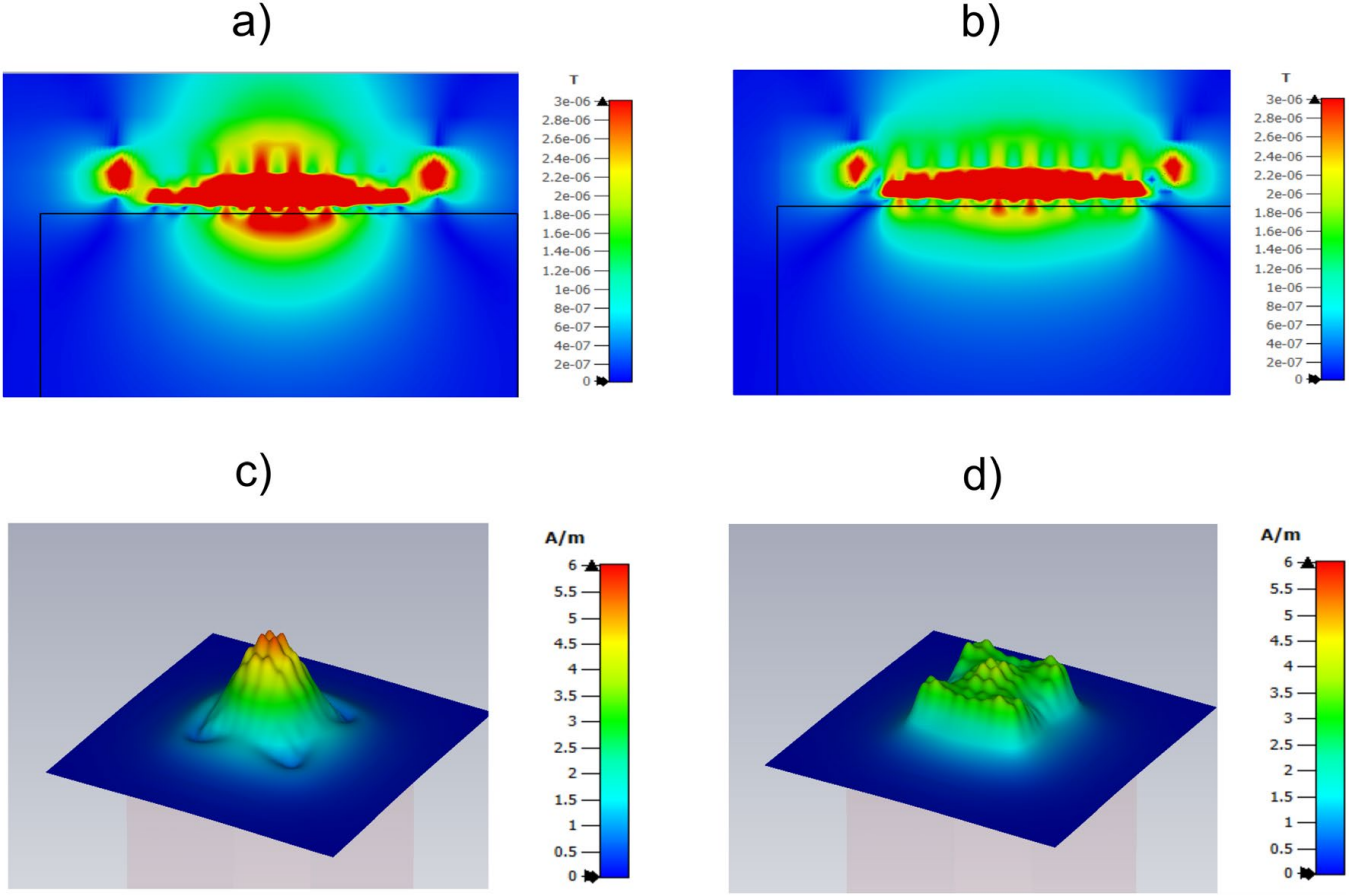
(**a**) RMS B$^+_1$ map obtained with Simulia CST in an axial plane for the squared coil of 12 in length loaded with a conducting sample and an array of $8 \times 8$ circular CLR with $a=12.$ mm, $r=6$mm, $w=2$ mm, $C=525$ pF. (**b**) RMS B$^+_1$ map obtained for the this same structure by changing from $C=525$ pF to $C=495$ pF the capacitors in the elements arranged at the edges of the array. (**c**, **d**) H maps for the above structures.

In the previous section, the ability of CLR arrays to enhance the SNR of surface coils has been demonstrated. As mentioned in the Introduction Section, CLR arrays can also be used to tailor the near-field pattern of a coil. In particular, in the present work, the possibility of making this near-field pattern more homogeneous at some distance is investigated. This is of practical interest for MRI since it can result in a more uniform image at a certain slice. As also pointed out in the Introduction Section, a more homogeneous field pattern can be achieved by preventing the reflection at the edges of the array of MI waves excited by the coil, so that a field pattern typical of traveling waves, which is more uniform, can be obtained. To avoid the reflections of the MI waves, in this work a remedy based on matching with a suitable load or terminal impedance at the ends of 1D arrays that support MI waves^32,33^ is extended to 2D arrays. The CLR arranged along the edges of the 2D array are matched by adding a terminal impedance that must account for the effect of missing neighbors beyond the boundaries of the array. This terminal impedance can be calculated from the mutual inductance between nearest neighbors. As mentioned above, for the array analyzed above with $a=12.5$ mm, the discrete model provides the following values for mutual inductance between the nearest neighbors in the same row, $M_\text {r}=-0.48$ nH, and on the same diagonal, $M_\text {d}=-0.13$ nH. At a frequency of 63.6 MHz, these mutual inductances provide the following reactances $X_\text {r}=\omega M_\text {r}=-0.19$ $\Omega$ and $X_\text {d}=\omega M_\text {d}=-0.05$ $\Omega$, respectively. For a CLR arranged at the edge of the array, the closest neighbors missing are one neighbor in the same row and two neighbors for two diagonals, so the total reactance contribution of the missing nearest neighbors is $X_\text {r}+2X_\text {d}=-0.29$ $\Omega$. This negative value is equivalent to the negative impedance value of a capacitor with a capacitance of 8.5 nF at 63.6 MHz. As mentioned, a capacitor with $C=525$ pF was used in the simulations carried out with Simulia CST and shown in Figs. 10 and 11. A 8.5 nF terminal capacitor connected in series with $C=525$ pF corresponds to an equivalent series capacitance of $C=495$ pF. It should be noted that the terminal impedance that matches the ends of the MI waveguides is, in general, a complex quantity given by the product of the mutual reactance and a complex phase factor^32,33^. In the present case, the real part or resistance is neglected to avoid the introduction of additional losses in the system that would degrade the SNR. Therefore, using a capacitance of $C=525$ pF for all CLR inside the array and $C=495$ for all CLR arranged at the edges of the array, the MI waves will be matched at the edges of the array and the reflections of the MI waves will be prevented. To verify this, Fig. 14a shows the $B_1^+$ map obtained with Simulia CST for the $8 \times 8$ array with $C=525$ pF in all CLR (and previously shown in Fig. 10b) to be compared with the $B_1^+$ map shown in Fig. 14b for $C=525$ pF in the CLR arranged inside the array and $C=495$ pF in the CLR arranged at the edges of the array. The $B_1^+$ map in Fig. 14b is more homogeneous, and the field value is still higher than the value provided by the coil itself (see Fig. 10a). Moreover, Figs. 14c and d show H field maps in a plane parallel to the array that make it clearer that the field is more uniform in the case of the array with suitable terminal impedances at the CLR arranged at the edges (Fig. 14d), as it is typical of a scenario with traveling waves and no reflected waves.

## Conclusion

The ability of MS made up of 2D CLR arrays arranged in a coplanar configuration to enhance the SNR of MRI surface coils has been demonstrated, which is of practical interest in MRI. CLR arrays introduce resonances in the frequency dependence of the input resistance $R_\text {in}$ of the coils due to the excitation of standing MI waves with an odd number of half-wavelengths that fit the size of the array. The SNR has been shown to be optimal at the frequency corresponding to the local minimum existing between the resonances in the frequency dependence of $R_\text {in}$. Moreover, it has also been demonstrated that operating at this frequency, the SNR can be enhanced if the mutual coupling between CLR in the array is made stronger by bringing closer the CLR to each other and by using CLR of squared shape instead of circular. These conclusions have been obtained in a study carried out with a fast and efficient algorithm developed for the numerical analysis of MRI coils loaded with CLR arrays and a conducting half-space resembling human tissue. The conclusions obtained from the results provided by this algorithm have been double-checked with numerical results obtained with the commercial electromagnetic solver Simulia CST and with experimental results. In addition, numerical results provided by Simulia CST have also been used to demonstrate that MS made up of CLR arrays can be used to tailor the near-field pattern of MRI coil, in particular, it is shown that a more homogeneous near-field pattern can be obtained by matching the edges of the array with suitable capacitors so that the reflection of backward MI waves at the edges is prevented. This allows to obtain more uniform MR images at certain slices, which is also of practical interest in MRI.

## Supplementary Information

Supplementary Information.

## Data Availability

The datasets used and/or analysed during the current study are included in this published article and its supplementary information files. All data generated or analysed during this study are available from the corresponding author on reasonable request.
