# Supplementary material for: Metasurfaces of capacitively loaded metallic rings for magnetic resonance imaging surface coils
Source: Sci Rep. 2023 Feb 21;13:2998. doi: 10.1038/s41598-023-30185-y (PMC10126161; doi:10.1038/s41598-023-30185-y)
Supplement: Supplementary file 1 — Supplementary Information. [file 41598_2023_30185_MOESM1_ESM.pdf]

Appendix: Linear relation between SNR and S_{21}

This Appendix demonstrates the linear relationship between the SNR provided by a surface coil and the transmission coefficient or scattering parameter S_{21} measured with a VNA between the coil matched to 50 Ω and a small probe. This relationship allows for comparative measurements of the SNR measured for a coil in the laboratory. In the general theory of transmission lines, the transmission coefficient or scattering parameter S21 for a two-port network is defined as[1]

$$S_{21} = \frac{V_2^-}{V_1^+}\Big|_{V_2^+=0}. \tag{1}$$

where V_1^+ is the voltage of the incident wave at port 1, V_2^- is the voltage of the reflected wave at port 2. The voltage of the incident wave at port 2, V_2^+, is zero, since both ports are matched to 50 Ω. The transmission line theory also establishes that the power associated with the voltage V_1^+ is $P = (V_1^+)^2/(2Z_0)$, where Z_0 is the characteristic impedance of the line. In the case of MRI surface coils, the transmission line corresponds to a coaxial cable with $Z_0 = 50\Omega$. The coil must be matched to an impedance of 50Ω for the right operation, and this is usually achieved by means of a matching network consisting of a shunt capacitor and a series capacitor. When the coil is set at 50 Ω, all power P is delivered to the input resistance R_{in} of the coil, that is, $P = R_{\text{in}}I^2/2$, where I is the amplitude of the current in the coil. The value of R_{in} accounts for the losses introduced into the coil by tissue, sample, or any other conducting element such as the CLR arrays analyzed in the present work. If the coil is connected to port 1, V_1^+ can be written as

$$V_1^+ = \sqrt{2Z_0P} = \sqrt{2Z_0}\sqrt{R_{\text{in}}I^2/2} = I\sqrt{Z_0R_{\text{in}}}. \tag{2}$$

Moreover, if port 2 is connected to a loop probe that is small enough so that it does not perturb the RF magnetic field produced by the coil, the voltage V_2^- induced in the probe by the magnetic field B produced by the coil is given by

$$V_2^- = -j\omega B_1 S, \tag{3}$$

where S is the cross-sectional area of the probe. After substituting V_1^+ y V_2^- in the definition of S_{21} it is obtained

$$S_{21} = \frac{V_2^-}{V_1^+} = -\frac{j\omega S}{\sqrt{Z_0}} \cdot \frac{B}{I\sqrt{R}} = -\frac{j\omega S}{\sqrt{Z_0}}\text{SNR}, \tag{4}$$

where it has been assumed that the SNR is given by the ratio $B_1/\sqrt{R_{\text{in}}}$, Therefore, the S_{21} between a small probe and a coil matched to 50 Ω is proportional to the SNR provided by the coil.

References

1. Pozar, D. *Microwave Engineering* (WileySon, 2011).
